# Acetabular fractures in the elderly treated with acute fixation and primary total hip arthroplasty: a 3-year follow-up of 70 patients

**DOI:** 10.1007/s00402-025-05941-6

**Published:** 2025-05-30

**Authors:** Anders Enocson, David Chang

**Affiliations:** 1 Karolinska Institutet, Stockholm, Sweden; 2 Karolinska University Hospital, Stockholm, Sweden

**Keywords:** Acetabular fracture, Elderly, Acute total hip arthroplasty, Geriatric trauma, Open reduction and internal fixation.

## Abstract

**Introduction:**

The number of acetabular fractures in the elderly population is increasing. Non-surgical treatment in these patients have been associated with poor outcomes. A primary total hip arthroplasty (THA) in combination with additional acetabular fixation (“fix and replace”) has been suggested to prevent complications associated with inactivity and to allow an active independent life. Although the reported results are promising, most of the so far published series are relatively small and with short follow-up times.

**Materials and methods:**

All patients aged from 60 years that underwent an acute primary THA with additional fixation (plate and/or cage) due to an acetabular fracture at the Karolinska University Hospital in Stockholm, Sweden from 2017 to 2023 were identified. Medical records including radiographs were manually reviewed and follow-up was a minimum of 1 year.

**Results:**

A total of 70 patients were included. The median age was 78 (60–95) years, and 26% (*n* = 18) were females. Six patients (8.6%) underwent an open reoperation due to infection (*n* = 4, 5.7%) or recurrent dislocations of the THA (*n* = 2, 2.9%). Four patients (5.7%) had a dislocation of the THA. The dislocation rate was 19% (*n* = 3/16) for THA via a posterior approach, and 1.9% (*n* = 1/54) for an anterolateral approach (*p* = 0.04). A total of 24 patients (34%) had at least one other adverse event. Logistic regression analysis showed that female gender was associated with an increased risk for other adverse events in both uni- (OR 4.7, 95% CI 1.5–15, *p* = 0.008) and multivariable (OR 5.3, 95% CI 1.6–18, *p* = 0.008) analysis. The 30-day mortality was 7.1% (*n* = 5), and the 1-year mortality was 13% (*n* = 9) for all patients.

**Conclusions:**

The reoperation rate and the mortality was moderate, whereas the rate of other adverse events was considerable. The posterior surgical approach was associated with an increased risk for dislocation.

## Background

The number of acetabular fractures in the elderly population is increasing in many countries [6, 16, 20]. Despite high age, many of these patients have high functional demands, and the treatment of choice needs not only to prevent complications associated with inactivity, but also to allow for an active independent life. Minimally displaced acetabular fractures (< 2 mm) can often be treated non-surgically, but due to poor bone quality many of the elderly patients present not only with severely displaced fractures, but also with comminution and irreversible damage to the cartilage of the hip joint [6, 8]. Non-surgical treatment in these patients have been reported to be associated with poor functional outcomes [2, 25], high secondary operation rates [19, 21] and increased mortality [3, 12, 15]. Primary surgical options include open reduction and internal fixation (ORIF), total hip arthroplasty (THA) or a combination of ORIF and THA [17], sometimes combined with an acetabular reinforcement cage/ring [5]. ORIF alone in elderly patients have been reported to be associated with a high rate of reoperations [2, 15, 17]. In addition, fracture fixation alone (ORIF) often means that the patient is restricted to limited weight-bearing of the affected leg during the fracture healing period (2–3 months). However, effective unloading using crutches or other walking aids is not possible for the majority of elderly patients, often resulting in inactivity or even bedrest. In recent years, there has been an increasing interest in treating elderly acetabular fracture patients with a primary THA in combination with ORIF and/or reinforcement cage (“fix and replace”). The indication is usually a multifragmentary comminuted fracture pattern and/or articular impaction [14]. Suggested advantages with this surgical approach are immediate full weight-bearing and good functional outcomes. Although the reported results are promising, most of the so far published series are relatively small and with short follow-up times [9, 13].

The aim of this study was to evaluate outcomes after acute primary THA with additional acetabular fixation in elderly patients with an acetabular fracture.

## Patients and methods

All patients aged from 60 years that underwent an acute (within 21 days) primary hip arthroplasty with additional fixation (plate(s) and/or cage) due to an acetabular fracture at the Karolinska University Hospital in Stockholm, Sweden from 2017 to 2023 were identified through the local surgical database. Patients with pathological or periprosthetic acetabular fractures were excluded. A letter with information about the study with the option to *opt-out* was sent to all patients. All medical records including radiographs were manually reviewed. Collected demographic variables included patient age, gender, ASA-class, fracture type and indication for surgery. Surgical variables including time to surgery, intraoperative estimated blood-loss, type of implants and surgical approaches were collected. Preoperative radiographs were analyzed by the two authors and the fractures were classified according to Judet and Letournele [11].

Follow-op variables included any surgical complication and reoperation, including cause and type, any adverse event not requiring surgical treatment (e.g. nerve injury, pneumonia, pulmonary embolism, deep venous thrombosis, urinary tract infection, sepsis, kidney failure, superficial wound infection). In addition, mortality at 3 months and 1 year was recorded. All patients were followed for a minimum of 1 year after the surgery.

The study was approved by the national Swedish authority for ethical approvals with reference number: 2024-06249-01.

### Statistical methods

Numerical data was presented as median (range). Categorical data was presented as frequency with percent distribution. Nominal variables were tested with the Fisher’s exact test. Logistic regression analysis was done to investigate potential factors associated with adverse events not requiring reoperations. At first, crude association for each variable was tested in univariable models. Subsequently, a multivariable model was used to study the adjusted associations. The associations were presented as odds ratios (ORs) with 95% confidence intervals (CIs). All tests were two-sided. The follow-up time was defined as the time from date of surgery to December 31, 2024 or death. The results were considered significant at *p* < 0.05. The statistical software used was IBM SPSS Statistics, Version 28 for Windows (SPSS Inc., Chicago, Illinois).

## Results

**Fig. 1:**
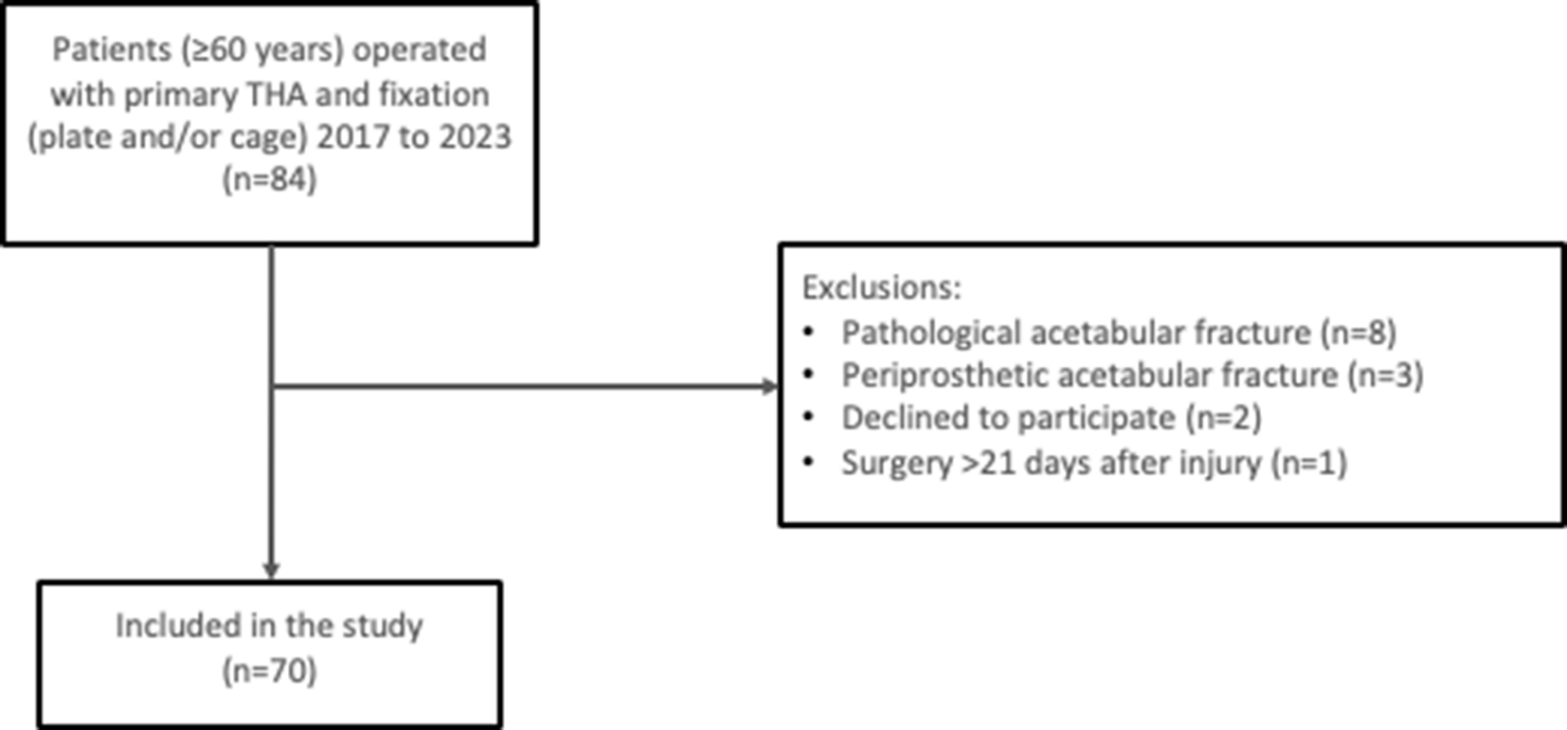
Flowchart of patient inclusion

**Fig. 2:**
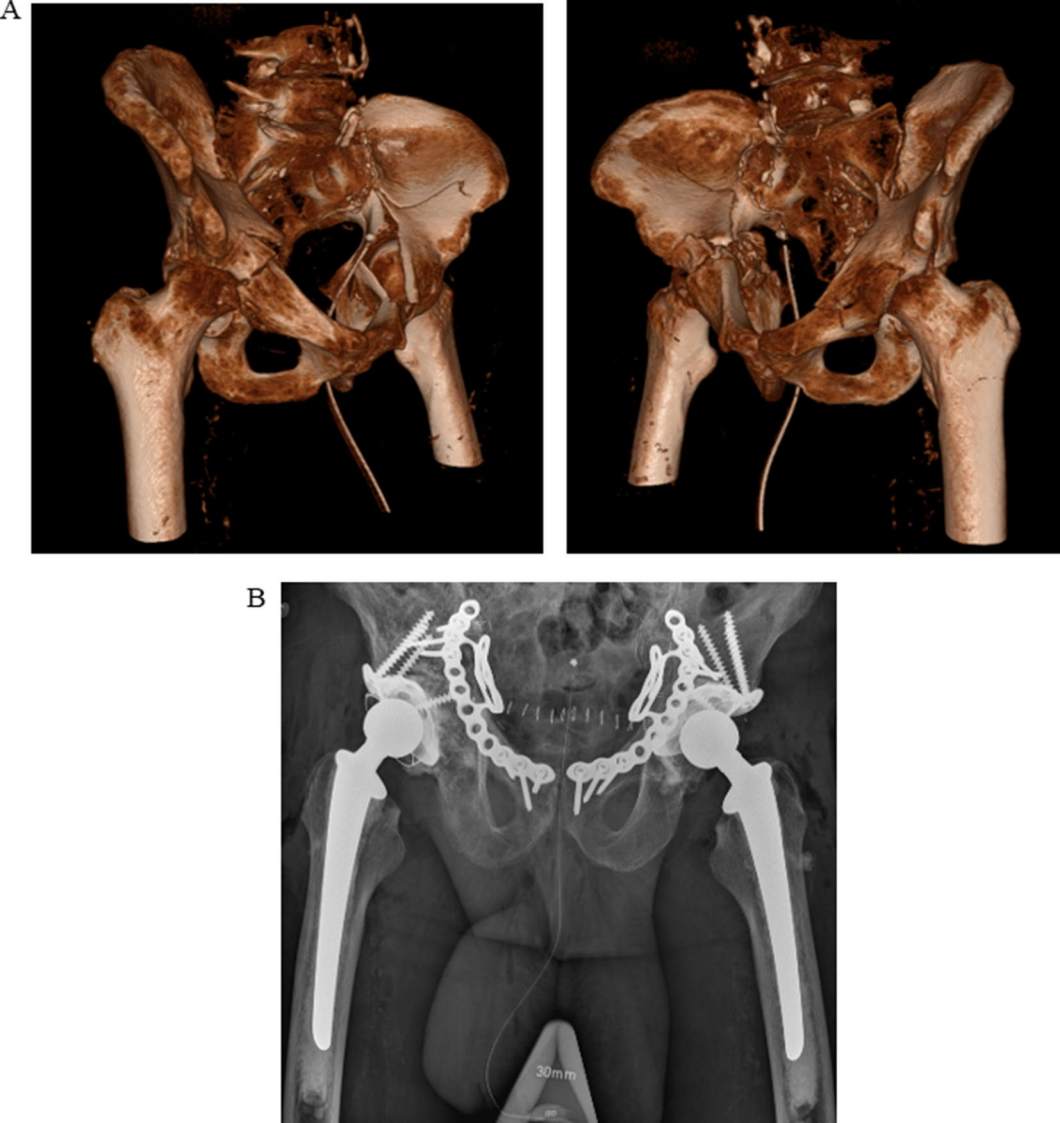
a-b. A 87-year old patient with bilateral anterior column + posterior hemitransverse fractures (**a**) treated with bilateral anterior plate, cage, autologous bone graft and THA (**b**)

A total of 84 patients were identified. After exclusion of 8 patients with a pathological acetabular fracture, 3 patients with a periprosthetic acetabular fracture, 2 patients who declined to participate (*opt-out*) and 1 patient who was operated 22 days after the injury, a total of 70 patients were included in the analysis (Fig. 1). Of those, 1 patient presented with bilateral similar fracture types, and was operated in both hips at the same time with the same type of implants and was therefore analyzed as one patient/case only (Fig. 2). The median age for all patients at the time of the operation was 78 (60–95) years, and 26% (*n* = 18) were females. The median age for females was 79 (60–87) years, and 78 (63–95) for males. The most common trauma mechanism was *simple fall* (76%, *n* = 53), followed by *fall from height* (11%, *n* = 8) and *traffic related injury* (11%, *n* = 8). The most common acetabular fracture type was *anterior column plus posterior hemitransverse* (39%, *n* = 27), followed by *associated both columns* (31%, *n* = 22) and *anterior column* (20%, *n* = 14). Four (5.7%) patients were admitted to the intensive care unit for 1–4 days. The median follow-up time was 1079 (6-2528) days (3 years). Additional data on patient, injury, and treatment characteristics are presented in Table 1.

**Table 1:** Patient, injury and treatment characteristics

Variable	All patients *n* = 70
Age; Median (range)	78 (60–95)
Gender Female; n (%)	18 (26)
ASA-class; n (%) 1 2 3 4	0 25 (36) 41 (59) 4 (5.7)
Injury mechanism; n (%) Simple fall Fall from height Traffic related Motorcycle related Other	53 (76) 8 (11) 8 (11) 14 (6.1) 1 (1.4)
High-energy trauma mechanism; n (%)	15 (21)
GCS; Median (range)	15 (3–15)
GCS < 9; n (%)	1 (1.4)
SBP on arrival (mmHg); Median (range)	137 (91–181)
Pulse rate on arrival; Median (range)	83 (50–150)
Hb on arrival (g/L); Median (range)	113 (76–154)
Head or neck injury; n (%)	2 (2.9)
Chest injury; n (%)	5 (7.1)
Abdominal injury; n (%)	0
Major spine injury; n (%)	3 (4.3)
Major upper limb injury; n (%)	4 (5.7)
Major lower limb injury; n (%)	2 (2.9)
Time to surgery (days); Median (range)	4.0 (1–21)
Indication for surgery; n (%) Dome impaction Posterior wall comminution	67 (96) 3 (4.3)
Hospital length of stay (days); Median (IQR)	5.0 (2–30)
ICU care; n (%)	4 (5.7)
ICU care length of stay (days); (range)	(1–4)

SD = standard deviation, IQR = interquartile range, ASA = American Society of Anesthesiologists, GCS = Glasgow Coma Scale, SBP = systolic blood pressure, Hb = hemoglobin, ICU = intensive care unit

## Surgical procedures

The median time from hospital admission to surgery was 96 (24–504) hours, and 49% (*n* = 34) were operated within 72 h. The median estimated intraoperative blood loss was 700 (200–3671) ml. All patients were operated with a cemented total hip arthroplasty with a Lubinus SPII stem (Waldemar Link, Hamburg, Germany) and a Lubinus SPII cup or a Marathon cup (DePuy Synthes, Richmond, Virginia, USA. All patients had additional fixation with reinforcement cage (97%, *n* = 68) and/or plating (67%, *n* = 47). The cages used were Müller (*n* = 63) or Burch-Schneider (*n* = 5; Zimmer Biomet, Warsaw, Indiana, USA). If necessary, autologous bone graft from the femoral head was impacted in the acetabulum. The most common implant combination was a THA with an anterior plate plus a cage (43%, *n* = 30; Table 2; Fig. 2). The THA and cage surgery was performed using an anterolateral [7] (77%, *n* = 54) or posterior (23%, *n* = 16) surgical approach, all in lateral decubitus position. All anterior plates (*n* = 35) were inserted via an anterior intrapelvic approach. Patients were operated by one of four experienced senior consultant orthopaedic surgeons trained in arthroplasty surgery as well as pelvic/acetabular fracture surgery. All surgeons were familiar with both posterior and anterolateral surgical approaches for arthroplasty surgery. Perioperative intravenous antibiotic prophylaxis was given to all patients, as well as postoperative low-molecular-weight heparin for prevention of blood clots. Postoperatively, patients were instructed to weight-bear as tolerated using adequate walking aids when needed.

**Table 2:** Acetabular fracture type in relation to type of acetabular fixation

	Type of fixation (*n*)						
Fracture type	Total	Cage + anterior plate	Cage	Cage + posterior plate	Cage + anterior plate + posterior plate	Anterior + posterior plate	Posterior plate
Anterior column + posterior hemitransverse	27	12	13	2	0	0	0
Associated both column	22	10	1	7	4	0	0
Anterior column	14	5	9	0	0	0	0
Transverse + posterior wall	2	0	0	0	1	1	0
Posterior wall	2	0	0	1	0	0	1
Unable to classify	3	3	0	0	0	0	0
Total	70	30	23	10	5	1	1

## Surgical complications and reoperations

Six patients (8.6%) underwent an open reoperation due to infection (*n* = 4, 5.7%) or recurrent dislocations of the THA (*n* = 2, 2.9%). The 4 patients reoperated due to infection presented within 14–35 days after the primary operation. Three of the patients with infection were successfully managed with antibiotics plus a single soft tissue debridement (*n* = 2) or two debridements (*n* = 1). One infection patient underwent 2 debridements including exchange of the femoral head. In addition, 3 patients had a postoperative deep infection that was treated non-surgically. One patient declined further surgery and the other 2 were judged to be too frail for additional surgery. These patients were treated with long-term antibiotics only. A total of 4 patients (5.7%) had a dislocation of the THA. Two of the 4 patients with dislocation were successfully treated with closed reductions only, whereas the other 2 patients underwent open revision surgery due to recurrent dislocations. The first dislocations occurred early in 3 patients (1, 1 or 23 days postoperatively respectively) and late in 1 patient (1.5 years). Three of the patients with dislocation were primary operated on using a posterior approach for the arthroplasty surgery, and 1 was operated on using an anterolateral approach. The dislocation rate was 19% (*n* = 3/16) for THA via a posterior approach, and 1.9% (*n* = 1/54) for an anterolateral approach (*p* = 0.04).

## Other adverse events and mortality

A total of 24 patients (34%) had at least one other adverse event; pulmonary embolism (*n* = 7, 10%), pneumonia (*n* = 7, 10%), urinary tract infection (*n* = 5, 7.1%), deep venous thrombosis (*n* = 3, 4.3%), sepsis (*n* = 3, 4.3%), nerve injury (*n* = 1, 1.4%) or stroke (*n* = 1, 1.4%). Logistic regression analysis was performed to evaluate potential risk factors for these other adverse events. Gender (female or male), ASA-class (ASA 1–2 or ASA 3–4), injury energy level (low-energy or high-energy) were tested. Female gender was associated with an increased risk for adverse events in both the uni- (OR 4.7, 95% CI 1.5–15, *p* = 0.008) and the multivariable (OR 5.3, 95% CI 1.6–18, *p* = 0.008) analysis (Table 3). The 30-day mortality for all patients was 7.1% (*n* = 5), and the 1-year mortality was 13% (*n* = 9).

**Table 3:** Logistic regression to evaluate factors associated with adverse events not requiring reoperation

Variable	Total	Adverse events	Univariable		Multivariable	
		*n* (%)	OR (95%CI)	*p*-value	OR (95%CI)	*p*-value
**Gender**						
Male Female	52 18	13 (25) 11 (61)	ref 4.7 (1.5–15)	0.008	ref 5.3 (1.6–18)	0.008
**ASA-class**						
1–2 3–4	24 46	5 (21) 19 (41)	ref 2.7 (0.9–8.4)	0.09	ref 3.1 (0.9–11)	0.08
**Injury type**						
Low-energy High-energy	55 15	20 (36) 4 (27)	ref 0.6 (0.2–2.3)	0.5	ref 0.8 (0.2–3.4)	0.8

OR = odds ratio, CI = confidence interval, ASA = American Society of Anesthesiologists, ref = reference

## Discussion

The main findings of this study were that the reoperation rate and the mortality was moderate, whereas the rate of other adverse events was considerable. In addition, the posterior surgical approach was associated with an increased risk for arthroplasty dislocation, compared to the anterolateral approach.

We found a moderate, or even low, reoperation rate (8.6%), due to infections or recurrent dislocations. In a study by Weaver et al., the reoperation rate was almost twice as high (14%) in 37 patients treated with primary THA and additional fixation [26]. As well as in our study, their main indications for reoperations were dislocations and infections. A similar reoperation rate was found in a large national register study with data from the United States by Upfill-Brown at el. They found a “revision-free survival” of 85% at 36 months, not specifying the indications for the reoperations [24]. In contrast, Manson et al. performed a prospective study comparing ORIF with ORIF plus THA. Interestingly, the patients could either choose the type of operation (ORIF or ORIF plus THA) themselves or choose to be randomized to either one of them. They did not use a cage, but a multi-hole revision porous coated acetabular component. In the end, 25 patients were treated with ORIF plus THA and the reoperation rate was 8.0% (*n* = 2), a number close to ours. No dislocations were reported, and it seems that various surgical approaches were used [17].

The rate of other adverse events was considerable in our series with one third (34%) of our patients having at least one. In addition, we found that female gender was a risk factor for these adverse events. In a systematic review by Jauregui et al. including 21 studies with 430 geriatric acetabular fracture patients treated with primary THA, the authors reported that 20% of the patients had a complication. The most common one being heterotopic ossification (20%), followed by dislocation of the arthroplasty (6.1%), venous thromboembolism (VTE; 4.1%) and infection (3.8%) [10]. In comparison, we had a much higher rate of VTEs, but similar rates of infections and dislocations. Furthermore, the dislocation rate in our series was much higher for the posterior surgical approach compared to the anterolateral (19% versus 1.9%). Interestingly, our numbers on dislocation rates were very close to the numbers reported in a previous series of 713 patients treated with a primary THA due to a femoral neck fracture, where patients operated with an anterolateral approach had a dislocation rate of 1.9%, compared to 13% after a posterior approach [4]. In our experience, an anterolateral surgical approach is well suited not only for arthroplasty surgery in these patients, but also for the implantation of a cage. Of course, in cases where posterior plating is done via a posterior (Kocher-Langerbeck) approach, this approach will be used for the arthroplasty and cage implants as well. Interestingly, we have not found any other studies that analyze the influence of gender on the risk for other adverse events in similar patient cohorts, although it has been described in pelvic fracture patients [1, 22]. We can only speculate about the reason(s) for our increased risk among female patients, also after adjustment for co-morbidity and injury type. It might have to do with hormonal differences, different ability to mobilize postoperatively or some other reason(s). As our sample size do not allow for further statistical analysis, we humbly only conclude that this topic clearly needs further investigation in future larger studies designed to answer this important question.

We found a 1-year mortality of 13%. A number which must be considered as moderate, or even low, in a cohort of geriatric patients with a median age of 78 years, a majority being ASA-class 3, that underwent major surgery. In a study by Stetzelberger et al., they compared mortality between surgically treated acetabular (*n* = 136) and hip fracture (*n* = 350) patients. They found an unadjusted 1-year mortality of 18% and 33%, respectively [23]. Similar mortality was reported in a recent study from Ljungdahl et al. including 148 geriatric patients with surgically treated acetabular fractures, whereof 54 with THA alone or THA combined with fixation. They found a 1-year mortality of 15% in these patients. In addition, they found about twice as high mortality (29%) among patients treated non-surgically [15]. They speculate that this difference in mortality between surgically and non-surgically treated patients might be due to patient selection. A reasonable interpretation that can be true for our patients as well. A further interpretation could be that the selection process was adequate as the patients treated non-surgically clearly were fragile, as demonstrated by their high mortality. On the other hand, we do not know if it was immobilization, pain and low functional outcomes that actually contributed to the high mortality in this patient group.

The implant combinations for fixation of the acetabular cup differs in the literature. We used a cage (reinforcement ring) in most cases, in combination with a cemented acetabular cup. In a systematic review by Zhang et al. including 33 studies, cages were used in about half of the reported series [27]. The optimal implant combination is yet to be determined, but it seems that several implant solutions result in satisfying results. Probably, a key factor for success in these complex operations is to use implants that the surgeon is familiar with. Common for most of the used acetabular implants is the use of screw-fixation of the cage or acetabular cup. In an interesting study by Marmor et al., they used 3-dimensional CT scans of 97 elderly patients with acetabular fractures to identify suitable corridors for screw fixation. They reported that 65% of the patients had at least 3 bone corridors available for screw fixation, and they concluded that a stable fixation of a cup/acetabular implant can be achieved in most geriatric patients [18].

## Strengths and limitations

A major strength of this study was the relatively large number of included patients. Another strength was the relatively long follow-up time, allowing for the capture of late as well as early reoperations and adverse events. This is underlined by the systematic review published by Zhang et al. including 33 studies, with still only 601 patients in total, and where the largest study comprised of only 55 patients, and with only 2 years’ follow-up time [27]. In addition, all reviewing of medical charts and fracture classification was performed by the two authors, assuring consistency in collecting the data. There were several limitations with the study, the major one being its retrospective design. Another limitation was the possibility that reoperations and other adverse events could have been missed if they were treated at other hospitals. However, since the Karolinska University hospital is the only hospital in the region treating these patients, the likelihood for this remains limited in an elderly population that is often quite resident. Another limitation was the lack of standardized follow-ups with functional outcomes. Still, if patients with cognitive dysfunction, severe frailty and even deceased patients are to be analyzes, this is difficult to perform without a large number of dropouts. The surgery investigated in this study can be characterized as highly complex, and therefore all patients were operated by one of only four surgeons at our clinic. Although they were all experienced and trained in both posterior and anterolateral surgical approaches for THA, it might still be a confounding factor if they were actually better at one of these approaches and thereby influencing the outcome.

## Conclusion

Our results support that an acute primary THA, preferably via an anterolateral surgical approach, in combination with additional acetabular fixation is a feasible option when treating elderly patients with acetabular fractures.

## Data Availability

The dataset used during the study is stored at the Karolinska Institutet and is available from the corresponding author upon reasonable request.

## Abbreviations

ASA: American Society of Anesthesiologists
CI: Confidence interval
GCS: Glasgow Coma Scale
Hb: Hemoglobin
ICU: Intensive care unit
OR: Odds ratio
ORIF: Open reduction and internal fixation
SBP: Systolic blood pressure
THA: Total hip arthroplasty
